# How to Predict Parturition in Cattle? A Literature Review of Automatic Devices and Technologies for Remote Monitoring and Calving Prediction

**DOI:** 10.3390/ani12030405

**Published:** 2022-02-08

**Authors:** Martina Crociati, Lakamy Sylla, Arianna De Vincenzi, Giuseppe Stradaioli, Maurizio Monaci

**Affiliations:** 1 Department of Veterinary Medicine, University of Perugia, Via S. Costanzo 4, 06126 Perugia, Italy; lakamy.sylla@unipg.it (L.S.); arianna.devincenzi@gmail.com (A.D.V.); maurizio.monaci@unipg.it (M.M.); 2 Centre for Perinatal and Reproductive Medicine, University of Perugia, 06126 Perugia, Italy; 3 Department of Agricultural, Food, Environmental and Animal Sciences (DI4A), University of Udine, Via Delle Scienze 206, 33100 Udine, Italy; giuseppe.stradaioli@uniud.it

**Keywords:** cattle, calving prediction, remote monitoring, calving assistance, calving alert

## Abstract

**Simple Summary:**

Cattle farming is progressively facing an increase in the number of animals that farmers must care for, resulting in decreasing time for observation of the single cow. A large amount of the scientific literature has been published concerning remote automatic devices and machine learning technologies for continuous monitoring of animal behavior and health status, including sensors for calving prediction This review summarizes the current status of the art concerning available automatic devices for the identification of the beginning of calving.

**Abstract:**

Cattle farming is facing an increase in number of animals that farmers must care for, together with decreasing time for observation of the single animal. Remote monitoring systems are needed in order to optimize workload and animal welfare. Where the presence of personnel is constant, for example in dairy farms with great number of lactating cows or with three milking/day, calving monitoring systems which send alerts during the prodromal stage of labor (stage I) could be beneficial. On the contrary, where the presence of farm personnel is not guaranteed, for example in smaller farms, systems which alert at the beginning of labor (stage II) could be preferred. In this case, time spent observing periparturient animals is reduced. The reliability of each calving alarm should also be considered: automatic sensors for body temperature and activity are characterized by a time interval of 6–12 h between the alarm and calving. Promising results have been shown by devices which could be placed within the vaginal canal, thus identifying the beginning of fetal expulsion and optimizing the timing of calving assistance. However, some cases of non-optimal local tolerability and cow welfare issues are reported. Future research should be aimed to improve Sensitivity (Se), Specificity (Sp) and Positive Predictive Value (PPV) of calving alert devices in order to decrease the number of false positive alarms and focusing on easy-to-apply, re-usable and well tolerated products.

## 1. Introduction

Good herd management is one of the major contributors to optimized reproductive performance and farm net return [1,2]. Calving monitoring and assistance represent a weak point worldwide; although sometimes neglected, parturition is a crucial event for both the dam and the newborn. Prolonged or difficult calving (dystocia) and untimed (both late and early) assistance can compromise welfare, fertility and milk production of the dam, together with survival, growth and future performance of the calf [3,4,5,6,7,8].

Dystocia is a great concern in dairy cattle, with an incidence ranging from 10.7 to 51.2% in USA, and from 2 to 22% in Europe. The variation can be associated with parity, breed, sire and sex of the calf [9,10]. In beef cattle, the incidence of difficult calving is usually lower and ranges from 3 to 7.7% [11,12]. Dystocia is recognized as a painful event, but pain evaluation and relief still need more attention among veterinarians and farmers [13,14]. Dystocia also results in substantial financial loss due to impaired absorption of immune-globulins from colostrum and to increased calf mortality [10,15]. Incidence of calf mortality within 48 h of life ranges from 5.3 to 13.2% in USA with the majority of events occurring in calves born from primiparous cows; in Australian beef pasture-based systems, it reaches 20% in primiparous dams [15,16,17].

Awareness of the effects of dystocia on dam and calf welfare, survival and farm net return is growing among farmers and stakeholders [5,18]. Improved calving monitoring and assistance are essential to timely recognition and resolution of dystocia and colostrum administration [19,20,21]. However, the identification of the exact beginning of parturition is challenging. The majority of farms rely on software to calculate the expected date of calving based on the day of the last insemination, but length of gestation varies. In systems where natural breeding is used or when the date of the last insemination was not recorded, the date of calving can only be presumed with approximation of 10 days [22,23,24].

**Figure 1:**
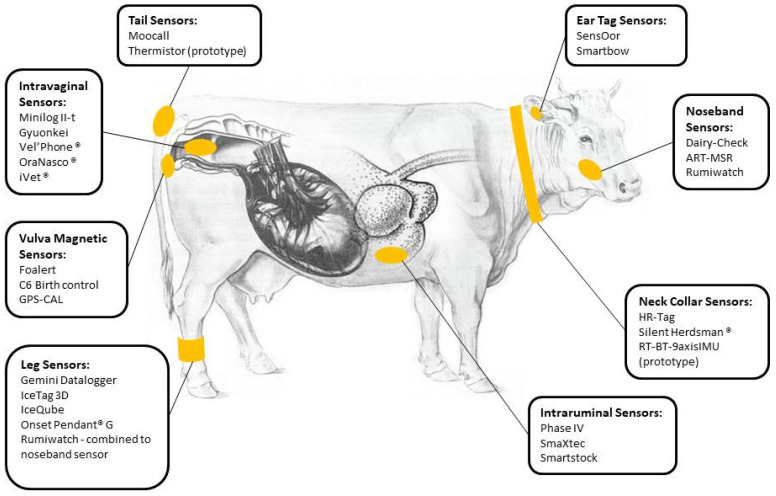
Application of available devices for calving prediction in cattle. Modified from: Richter and Götze (1978), Fig. 100, pp 142 [22].

This review summarizes the available methods, including “wearable” sensors, dedicated algorithms and machine-learning technologies for calving prediction. Figure 1 shows various possibilities for sensors placement on the cattle’s body, together with the main devices available from the industry. Table 1, Table 2, Table 3, Table 4 and Table 5 show devices currently available for imminent calving detection, with relative descriptions of time interval of delivery prediction, performance and manufacturer information when available.

Only products and prototypes described in the scientific, peer-reviewed literature were considered.

## 2. Pre-Calving Variation in Feeding, Activity, and Temperature

Pre-calving changes in behavior in cattle are represented by increased restlessness, reduced feed intake and rumination, seeking for isolation associated with frequent postural changes, tail raising and greater frequency of lying bouts [26,28,63,64,65,66,67]. Those changes in behavior become more frequent in the last hours before calving (see reviews by Saint-Dizier and Chastant-Maillard [68] and by Chang et al. [69]). Therefore, various approaches to automatic detection in order to predict parturition have been developed.

Visual observation of periparturient animals could be carried out through video recording by cameras placed on the maternity pen, but this method is time-consuming and rarely used [26,70]. The frequent presence of an observer could also induce discomfort in periparturient animals, induce the release of catecholamines and interfere with the calving process [3]. Cangar et al. [71] developed an algorithm for the automatic real-time monitoring of locomotion and posture of periparturient cows based on online image analysis through video cameras. The algorithm correctly classified 85–87% of behaviors when compared to the evaluation provided by operators. automatic algorithms for image analysis could represent a worthy-to-investigate approach for the identification of parturient animals in the future.

Researchers tried to commute the evaluation of premonitory signs of calving into an equation in order to predict the exact beginning of the expulsive phase, that is characterized by intense uterine contraction and expulsion of the fetus [72]. For example, Lange et al. [73] used a general linear mixed model to evaluate the relationship between observed tail raising, stepping, clear or bloody vaginal discharge and lateral lying associated with abdominal contractions in late gestation dairy cows on effective moment of calving. They used the estimates from the model to build the following equation for calving prediction:

Hours until parturition = 97.99 + (tail raising × −38.0) + (stepping × −37.65) + (clear vaginal discharge × −25.78) + (bloody vaginal discharge × −51.88) + (lying lateral with abdominal contractions × −30.52).

However, the same authors strongly recommended the observation of late-gestation dairy cows every two hours, which could negatively affect the possibility of field application.

Studies on body temperature identified a, decrease of 0.2–0.4 °C in vaginal and rectal temperatures at two consecutive measurements, 36–24 h before delivery [74,75,76]. According to those studies, vaginal and rectal temperature showed Se = 62–71% and Sp = 81–87%, and Se = 44–69% and Sp = 86–88%, respectively, for calving prediction.

## 3. Wearable Sensors for Automatic Monitoring: What Can We Measure?

The most frequently used sensors in cattle farming are pedometers and accelerometers, originally designed for estrus detection, followed by collar microphones for feeding and rumination control, and thermometers. Those sensors can be applied in different areas of the cow’s body in order to collect data on tri-axial movements and/or sounds (legs, neck, ears or tail, see Figure 1). Studies have shown that automated sensors are reliable, when compared to visual observation [77]. Parameters analyzed include rumination time, standing time, number of steps, lying time and number of lying bouts, tail raising or a combination of those, which are used to build a baseline. Deviations are indicative of parturition in the following 6–12 h [67,69]. However, when used as predictors of calving, some refinement to the algorithm should be considered; some authors reported that tail raising, standing and lying bouts increased in frequency 4 h before calving in primiparous and 2 h before delivery in multiparous Holstein cows [65,78,79,80,81]. Proudfoot et al. [25] observed that cows with dystocia displayed greater number of standing bouts 24 h before calving, compared to cows with eutocia (threshold: 33.8 bouts/day; Se = 77.8%; Sp = 77.8%). Moreover, behavioral changes could also be influenced by genotype; a periparturient Friesian cows show more stepping and walking activity than Jersey and Crossbred cattle [82]. A rumination sensor fixed to a noseband (ART-MSR; Agroscope Reckenholz-Tänikon, Ettenhausen, Switzerland) was created for experimental purpose, by Nydegger et al. [83], and used by Pahl et al. [35]. They found that cows stopped ruminating on average 123 ± 58 min before the onset of calving. An halter applied to the masseter muscles could be used to check rumination for calving prediction, but this prototype is not currently used [34].

Using commercially available microphones to monitor chewing, Schirmann et al. [84] and Calamari et al. [85] observed that cows spent on average 70%, or 63 ± 30 min/24 h, less time ruminating in the 24 h period prior to calving. In their observation feeding time also decreased by 66 ± 15 min, while dry matter intake declined by 56% in the last 6 h before calving, compared to a baseline. These findings could be useful for setting algorithms for calving alerts in future applications.

In other ungulates, such as caribou (*Rangifer tarandus caribou*), telemetric data from pregnant females were collected through a GPS radio collar (model G2110E, Advanced Telemetry Systems—ATS, Isanti, MN, USA) and have been used to identify the moment of parturition with an accuracy of 97%, and to assess neonatal survival (73% accuracy), based on dam movement pattern [86]. This methodology is not currently used in cattle, but grazing-based systems offer a field of application in the future. A modified tri-axial accelerometer commercially available in the form of ear-tag (Smartbow GmbH, Weibern, Austria) was modified for experimental purpose and used as a tail raising sensor in five at-term dairy cows for the prediction of imminent calving [41]. Due to the limited number of animals involved, these authors recommended more trials before applying this accelerometer as a calving predictor under field condition.

Automatic devices for continuous measurement of body temperature could be used for calving prediction: vaginal [29,87] and ruminal [43,44] sensors are able to identify a temperature drop from 0.3–0.5 °C as predictive of calving in the next 48–72 h. However, one drawback of the ruminal device is that once placed into the forestomach, the sensor cannot be re-used in other parturient dams. A temperature and pH intra-rumen sensor (SmaXtec Animal Care GmbH, Graz, Austria) has been used by Kovács et al. [45]. They reported a decrease in rumen temperature approximately 20 h before calving (0.48 ± 0.05 °C) in cows with normal delivery, while in animals with dystocia, temperature dropped 32 h before parturition (0.23 ± 0.02 °C). More recently, a thermistor (Prototype: 103JT-025, SEMITEC Corporation, Tokyo, Japan) for the continuous measurement of the tail skin temperature has been evaluated in adult cows for prediction of calving [46,47], and successively implemented through machine-learning algorithms [48]. Calving within the next 24 h was successfully predicted by the algorithm, but at the best of authors knowledge, this sensor is not commercially available.

Moocall (Moocall Ltd., Dublin, Ireland) is an inclinometer-accelerometer specifically created to fit the tail of cows and for monitoring tail movements in order to predict parturition [51]. Significant deviation from the hourly calculated baseline in tail movements lead to calving alarm, which was sent to operators by GSM. Different studies validated this product in field conditions: calving could be predicted in the next 24 h with Se = 100% and Sp = 95%, and with Se = 94% and Sp = 77% in a time-window of 3 h. Voß et al. [52] reported Se and Sp declining from 75 to 19% and from 96 to 63%, respectively, and positive predictive value (PPV) from 56 to 12%, for delivery prediction in 24 and 1-h intervals, respectively. Horváth et al. [31] reported PPV of 12.6%, with greater number of false positive alerts in primiparous compared to multiparous cows (*p* < 0.05). Some animals showed low skin tolerance of the device. A similar prototype which is fixed to the tail by elastic bandage wrapping is under development in Ireland [88].

### 3.1. Combining Data from Activity, Feeding and Other Behaviors

In the last decade, research has focused on merging data concerning different behavior or deriving various monitoring devices to improve the prediction of calving.

RumiWatch is a device consisting of a combination of a noseband-sensor and a hind limb accelerometer (Itin + Hoch GmbH, Fütterungstechnik CH-4410, Liestal, Switzerland) for rumination and activity measurements. Prediction of parturition in the next 3 h was achieved with a slight difference for multiparous and primiparous cows, as shown in Table 2 [36,37]. However, the authors found a relatively high number of false positive alarms, which could negatively affect the suitability for their application in field practice.

Data recording ear tags were recently implemented on the market and allow the continuous collection of information such as cow activity, feeding, rumination and ear temperature on an hourly basis. This aspect could be particularly valuable for the prediction of general health status, estrus detection and also for calving behavior. Rutten et al. [38] evaluated an algorithm for delivery prediction within one hour interval. They reported that sensitivity was too low (Table 2) while increasing the time window to 12 h led to improvement in sensitivity but to an even greater risk of false alarms. Using a time window of 1–3 h Krieger et al. [39,40] achieved a good performance (Table 2). The authors interestingly reported that cows which required assistance at parturition, showed a greater number of position transition events from −4 to −2 h before calving, compared to cows which required no assistance. Data characterizing this difference could be used for future improvement of the algorithm in order to differentiate animals at risk of dystocia and thus optimizing workload and periparturient cow monitoring.

### 3.2. Performance of Automatic Sensors and Machine-Learning for Remote Calving Prediction

The main advantage offered by automatic sensors is ensuring continuous data flow into farm management software with no additional farmer workload. As previously stated, commercially available devices are mainly designed for estrus detection. However, manufacturers offer additional updates for management software which include data analysis for calving prediction. Table 1, Table 2 and Table 3 show the most common products, with data concerning their performance in experimental field conditions. Accelerometers such as IceTag 3D and IceQube (IceRobotics, Edinburgh, UK), Onset Pendant^®^ G data logger can be fixed to neck collars or hind legs, while devices such as SensOor (Agis Automatisering BV, Harmelen, The Netherlands) are fixed to the ear identification tag to monitor rumination. They are able to send an alert for an incoming delivery with an uncertainty interval of at least 12–6 h [26,28,29]. Benaissa et al. [89] tried to reduce the interval from alert to calving at 4–2 h by combining data from three sensors: two accelerometers designed for human movement studies which were placed on the hind-leg and neck-collar of cows (Axivity AX3 loggers, Axivity Ltd., Newcastle, UK), and a localization sensor fixed to the neck-collar (DecaWave, Dublin, Ireland). However, trials revealed low Precision (Pr) and Se (67–79% and 63–69%, respectively) for use in the field. Refinement through machine-learning algorithms showed a better performance as reported by Miller et al. [32,33]. They used merged data from neck-collar accelerometers (Silent Herdsman^®^ SHM collars, Afimilk Ltd., Afikim, Israel), and from a tail-mounted tri-axial accelerometer (AX3, 3-Axis logging accelerometer; Axivity, Newcastle, UK) in both dairy and beef cattle. Tail rising event was the most important parameter for calving prediction, and calf expulsion could be predicted in a time-window of 5 h with slight difference in dairy and beef cattle (Se = 78.6%; Sp = 83.5% and Se = 76.1%; Sp = 83.3%, respectively).

Collar microphones such as HR-Tag and Hi Tag (HR-Tag, SCR Engineers Ltd., Netanya, Israel), showed a Se ~70% and Sp ~70% in predicting calving in the next 24 h [27,30,84]. Horváth et al. [31] applied a threshold of 10% decline in rumination, compared to the single cow baseline, and successfully predicted calving with 2–4 h interval.

One of the most recently introduced technique for big data analysis in livestock farming is represented by machine-learning and deep-learning algorithms. Briefly, software can be taught how to analyze a database and self-correct, thus improving the prediction of a certain event. An inertial measuring unit (RT-BT-9axisIMU, RT Corporation, Tokyo, Japan) has been applied as a neck collar to detect changes in activity in prepartum Japanese Black beef cattle. Data collected were then elaborated through an innovative Long Short Time Memory-Recurrent Neural Network (LSTM-RNN) model [42]. Similarly, the combination of deep-learning and machine-learning algorithms to analyze variation in activity, lying, standing, inactivity time, eating and rumination in dairy cattle lead to the prediction of calving within the next 3 h with a Se = 57%, Sp = 85% and Pr = 0.49% in the study conducted by Liseune et al. [90]. Reducing the time interval for calving prediction negatively affected the performance of the algorithm. Researchers used 360-degree cameras placed above the calving barn to extract behavioral data form video recordings and analyzed them through the Hidden Markov Model and the Viterbi algorithm [91], or the Adsorbing Markov Chain Model [92], to predict calving in dairy cows. In the first trial on 10 primiparous, the algorithm successfully predicted calving with Se = 91.05% and Pr = 93.28% and an overall Accuracy (Acc) ~91%. The Adsorbing Markov Chain Model successfully reduced the interval between calving prediction and calving event to 3 h in 25 Holstein and Brown Swiss cows. Another study reported an accurate prediction of the day of calving but not the exact hour [93]. Generally, authors agree that algorithms correctly classify data on eating, ruminating, lying and standing, but they should be refined before extensive application for precision calving prediction.

These results are encouraging and surely open a perspective for future applications that include analysis of data from video recordings and sensors to predict parturition with increasing accuracy.

### 3.3. General Considerations on Feeding, Activity and Temperature Remote Monitoring Devices Used for Calving Prediction

Differences between studies in baseline and differential rumination time, lying time and lying bouts around calving could be due to factors such as variation in dietary fiber content and housing systems. Moreover, it should be considered that sensors which rely on activity and/or behavior (tail rising, aimless walking) to identify calving could be biased by variation on the individual animal basis. Even though the majority of cows show increased lying bouts as calving approaches, for example, a certain percentage of subjects show no changes at all [69,94].

Overall, refinement of technologies for continuous remote monitoring of cow activities such as neck-accelerometers and ear tags accelerometers provide a new opportunity to predict calving time. The major advantage of these systems is represented by their application for general health monitoring (rumination), estrus detection (accelerometers). Thus, they are often already implemented in Precision Livestock Farming context, and the specific function for calving prediction could be achieved by software updating by the producers. The cost for purchasing this additional functionality or for sensors substitution should be considered.

The aforementioned methods and instruments are easy to apply, but they do not allow the identification of the exact onset of calving. The time interval between the alert and parturition varies from 24 to 6 h, thus they could be currently useful to identify the prodromal stage and to warn the farmer when to move cows to the maternity pen. As a general rule, the more the time window for alerts is reduced, the greater the risk of false alarms. This aspect inevitably affects the farmer willingness to use these devices. Concerning the prodromal phase (stage I) of calving, this is characterized by subtle physical modifications as an increase of the myometrium contractions or endocrine patterns (P4 decrease and oxytocin and PGF_2α_ increase). Sensors able to records these changes are still unavailable or too complicated and expensive [95,96].

Moreover, it should be noted that precision of those systems could also be affected by conditions other than calving, such as fever and lameness, housing and daily farm events [31,97]. When the identification of the exact beginning of expulsive phase is necessary, direct and regular observation of periparturient animals is still needed. Since those devices are able to identify changes in behaviors which occur during the prodromal (preparatory) stage of labor [72], farmers could rely on those warning to decide when moving cows in dedicated maternity areas and to intensify the surveillance of some specific animals.

## 4. Devices Which Identify the Stage II of Labor

The expulsive phase is characterized by the complete dilation of birth canal, fetal sacs rupture, fetus entering the canal together with intense and coordinated uterine and abdominal contractions [72]. Sensors for the detection of the stage II of labor can be divided into two main categories: external devices which are sutured to the vulvar skin, and intravaginal sensors.

### 4.1. Vulvar Magnetic Sensors

Vulvar lips separation during labor can be detected through magnetic sensors which are sutured to the vulva skin, as routine in equine practice (Foalert, Acworth, GA, USA; C6 birth control, Sisteck s.r.l., Sassuolo, Italy) [53,98]. Marchesi et al. [55] evaluated Se and PPV of Foalert as a calving alarm in 53 Holstein Friesian cows, which were found to be 100% and 95% respectively. They also reported that the presence of farm personnel at calving reached 100% in alarmed cows, compared to 17% in controls (*p* < 0.001). This system has been further paired to a GPS-transmitter included in neck collars of grazing dairy and beef cattle [56]. In grazing systems, GPS localization and calving alert could be useful both for ensuring assistance, first neonatal care, and to avoid calf losses due to predation. Although those devices proved to be suitable for correct identification of parturition in cattle, the application of the device is invasive and requires veterinarian supervision due to the necessity of a local anesthesia for the suture of the components to vulva surface. This technology has been evaluated as expensive for its application in cattle breeding operations, due to purchase and maintenance costs [16,54].

### 4.2. Intravaginal Devices

Some intravaginal thermometers are able to recognize both the pre-calving drop in dam’s body temperature and their own expulsion when fetal sacs or the fetus enter the birth canal. The T-shaped calving alert iVET^®^ (iVET^®^—Geburtsüberwachung für Kühe, 2012) is characterized by a light-sensor and has been evaluated for use in Holstein primiparous [61]. Sensitivity (Se) and specificity (Sp) of the iVET^®^ were 0.78 and 0.93, respectively. Although the device correctly warned the farm personnel, poor local tolerance was observed, as irritation and discomfort were noticed. Moreover, interference with the parturition process was hypothesized, as the shape of the device could be responsible for premature rupture of fetal sacs, delayed birth canal dilation and increased dystocia rate (58.3% and 40.9% in experimental and control primiparous, respectively, *p* < 0.001).

The Medria (Vel’Phone^®^, Châteaugiron, France) and the Gyuonkei (Gyuonkei, Remote Inc., Oita, Japan) are temperature sensors able to generate both an alert at approximately 24 h before delivery (decrease of 0.4 °C of vaginal temperature), and a calving alarm when the devices are expelled [31,49,50,62]. However, one of the major concerns when using devices only equipped with thermometers is that the differential between the dam’s temperature and the external environment could be not enough to generate the alert, as introduced by Norman et al. [16]. They described the use of a remote calving alert in deer, elk, bison and antelope (Sirtrack Ltd., Havelock North, New Zealand), but the detection of expulsion could be impaired for example in hot-climate conditions, as in cattle in case of heat stress.

The intravaginal device OraNasco (Kronotech Srl, Campoformido, Italy) overcomes the single-parameter issues since it is equipped with physical sensors for both light and temperature. The temperature sensor is set to recognize gradients. The light sensor is able to generate an output even in case of scarce brightness. When the device is inserted into the vaginal canal, the probe detects light or a sudden change in temperature. If at least one of the two conditions is present, the probe switches to the ejected status and communicates the expulsion to the Central Unit. The expulsion of the probe occurs when the fetal sacs or the fetus itself enter the birth canal, at the beginning of stage II of labor. Then, the Central Unit sends alerts to farm personnel through GSM, LAN and Wi-Fi connection. The remote system has been evaluated for use both in cattle [57] and buffaloes [58]. Field trials demonstrated an overall Se = 86.3% [60], a good local tolerance and a high retention rate, except in one case of recurrent vaginal prolapse in a buffalo heifer.

Watanabe et al. [99] evaluated the potential of an intravaginal device composed of a triaxial accelerometer coupled to a continuous radio-emitting body. Once expelled, the radio signal is no longer dampened by the body tissues, while the accelerometer identifies the falling. The combination of those data is commuted into the calving alarm by the Central Unit. The identification of stage II of calving was reported to occur correctly for both the triaxial and radio signal methods, although no further information is available concerning the local tolerance or field use of this device.

The majority of the products described above are not designed for use in grazing herds, as the central unit is to be placed within a range from periparturient cows. Due to the dispersal of herds in extensive Australian pasture-based systems, a telemetric intravaginal calving alert device is under evaluation in beef cattle [100]; the device is equipped with a Taggle^TM^ mother board which emits a radio signal ping when expelled. The radio ping can be telemetrically triangulated and the position localized within the pasture. Preliminary results showed an 85% retention rate and no local adverse effects. Correct identification of calving was reached in 66% of deliveries while localization of animals was achieved in 64% of cases with an approximation of 100–200 m. Tracking parturient animals in extensive grazing areas is a concern in Australian breeding systems. Placing radio receiving antennae could be difficult due to the ground topography; thus researchers are also evaluating a Vaginal Implant Transmitter (VIT) device which is equipped with temperature and accelerometer sensors coupled to a GNSS collar for tracking via satellite technology [101]. The device is still a prototype at present, and more improvement is needed for field use.

### 4.3. General Considerations on Devices for the Identification of Stage II of Calving

The methods included in this section are able to precisely identify the beginning of the expulsive phase, thus warning farm personnel and encouraging timely intervention. The duration of stage 2 of calving was reported to average 64.0 min for unassisted primiparous, 42.7 min for assisted primiparous and 20 min in multiparous cows [79]. Methods for the identification of the beginning of expulsive phase generate phone alerts and relative time of alarm reception could be used to schedule intervention in case time interval from alert and calving progression exceeds the median duration of stage II [102]. Those methods are exclusively dedicated to calving prediction. This means that they could not be used for multiple functions, such as estrus detection, rumination monitoring, nor to decide when moving cows to the maternity pen. The purchase of this technology should be evaluated considering the possibility to re-use the device for multiple cows and the return of improved calving management. Moreover, in smaller herds, where the number of employees could not ensure continuous monitoring of periparturient cows, the presence of personnel within the calving barn is optimized and time spent monitoring animals is reduced. Specific employment in farms where the value of the calf is relevant (sorted semen, embryo transfer), can also benefit from these technologies. Timely calving assistance is beneficial for the overall farm reproductive and productive outcomes [102]. Palombi et al. [57] demonstrated that timely calving assistance and initial neonatal care reduce the incidence of postpartum uterine diseases such as retention of fetal membranes, metritis and neonatal mortality. Decreased incidence of uterine infections led both to reduced calving-conception interval and number of artificial inseminations per pregnancy in monitored dairy cows.

Ensuring colostrum intake during the first 6 h of life is fundamental for calf survival and welfare [20]; Morin et al. [103] found a positive association between adequate transfer of passive immunity and first colostrum feeding before 3 h of life. However, they also observed that only 42% of the newborn calves receive their first meal within this time interval and recommended farmers to improve calves and colostrum management. Therefore, increased workload for calving monitoring and newborn calf care could be perceived by farmers as time-consuming and expensive, but partial budget estimation of the effect of calving monitoring and assistance confirmed that a 100-lactating dairy herd could improve the net return from 37 to 90 €/cow/year. Those incomes resulted from reducing calf losses, involuntary culling during the first 60 days postpartum, and days open associated with an increased milk yield [59]. Consequently, investing in calf care could be paid back through the increased number of weaned calves for selling or replacement. On the other hand, exact identification of stage II of parturition could be beneficial in case early cow–calf separation and pathogen-free colostrum feeding is mandatory for the eradication of vertically transmitted diseases such as paratuberculosis and bovine leukemia virus [104,105].

## 5. Conclusions

Prediction of parturition based on continuous visual observation, hormone assay and manual temperature monitoring is time consuming, expensive and not realistically achievable in the majority of intensive breeding systems. Thus, remote monitoring and automated warning systems for health management, including parturition, are needed in order to optimize workload, animal welfare and farm net return. Farm managers should take into account the need to precisely identify the beginning of labor and the costs when choosing an automatic monitoring system based on sensors, rather than purchasing dedicated calving alarm devices. Where the presence of personnel is constant, such as on farms with a great number of lactating cows or with three milkings/day, calving monitoring systems which send alerts during the prodromal stage of labor (6–12 h before) could be beneficial. In this scenario, observation of periparturient cows could be carried out at regular intervals across the farm routine, without increasing working-hour costs. On the contrary, when the presence of farm personnel is not guaranteed, systems which alert at the beginning of labor could be preferred. In this case, time spent observing periparturient cows is reduced and dedicated intervention at the moment of delivery will decrease personnel-related costs.

Another relevant aspect is the reliability of each calving alarm: automatic sensors for body temperature and activity are largely used in cattle farming in order to monitor health and could be also adapted as imminent calving detectors. Even if most of them are characterized by good Se and Sp, increasing sensitivity could lead to excessive number of false positive alarms, which is detrimental on farmer willingness to use them. Moreover, it should be remarked that precision of those systems could also be affected by conditions other than calving, such us fever and lameness, cow comfort and housing, flooring and daily farm events. Promising results have been shown by devices which could be placed within the vaginal canal, thus identifying the beginning of fetal expulsion and optimizing the timing of obstetric assistance, even if some cases of non-optimal local tolerability are reported.

Future efforts should be aimed to improve Se, Sp and PPV of calving alert devices, decreasing the number of false positive alarms and prioritizing easy-to-apply, re-usable and well tolerated products.

## Figures and Tables

**Table 1:** Devices for automatic activity and feeding monitoring for calving prediction, with performance and references.

Parameter	Sensor Type	Device	N	Application	TI	Device Performance	Factory	References
Activity and leg position	Accelerometer	Gemini Datalogger	101	Hind leg	24 h ^1^	Se = 77.8%; Sp = 77.8%; Acc = 77.8% ^1^	Gemini Dataloggers Ltd., Chichester, UK (NS)	Proudfoot et al. [25]
		IceTag 3D	38	Hind leg	6 h	n.a.	IceRobotics Ltd., Edinburgh, UK http://www.icerobotics.com	Jensen [26]
		IceQube	132	Hind leg	6 h 14 min (range: 2 h–14 h 15 min) >4 h in 76% of cows	n.a.	IceRobotics, Ltd., Edinburgh, UK http://www.icerobotics.com/	Borchers et al. [27] Titler et al. [28]
		Onset Pendant^®^ G	42	Hind leg	24 h	Se = 58%; Sp = 58%; PPV = 34%; NPV = 79%; AUC = 0.60	Onset Computer Corporation, Bourne, MA https://www.onsetcomp.com/products/data-loggers/ua-004-64/ (NS)	Ouellet et al. [29]
					12 h	Se = 52%; Sp = 54%; PPV = 15%; NPV = 88%; AUC = 0.56		
					6 h	Se = 58%; Sp = 61%; PPV = 10%; NPV = 95%; AUC = 0.61		
Eating and rumination	Microphone/ accelerometer	HR-Tag	27	Neck collar	24 h	Se ~70%; Sp ~70%	SCR Engineers, Ltd., Netanya, Israel https://www.allflex.global/	Clark et al. [30]
		Ruminact™ Hr-Tag	54	Neck collar	From 4 h to 2 h	n.a.	SCR Engineers, Ltd., Netanya, Israel https://www.allflex.global/	Horvàth et al. [31]
	Accelerometer	Silent Herdsman^®^ SHM ^2^	110	Neck collar	5 h	Dairy cattle Se = 66.7%; Sp = 62.3%; AUC = 68.2%	Afimilk Ltd., Israel www.afimilk.com/	Miller et al. [32]
			144			Beef cattle Se = 70.9%; Sp = 71.5%; AUC = 78.1%		Miller et al. [33]
	Elecromyography	Dairy-Check	17	Noseband	6 h	n.a.	BITSz Engineering GmbH, Zwickau, Germany http://www.bitsz-electronics.de/ (NS)	Büchel and Sundrum [34]
	Pressure	ART-MSR	17	Noseband	2 h	n.a.	ART-MSR; Agroscope Reckenholz-Tänikon, Ettenhausen, Switzerland https://www.msr.ch/en/product/special_data_logger/rumination_sensor/ (NS)	Pahl et al. [35]

N: number of animals; NS: not specific, means that devices was self-assembled by researchers starting from data logger and sensor available in the market. This means that there are no commercially available products specific for use in cow. TI: time interval between calving alarm and parturition. Se: sensitivity; Sp: specificity; Acc: accuracy; PPV: positive predictive value; NPV: negative predicting value; AUC: area under curve; n.a.: not available. ^1^ The experiment was designed for the identification of cows with dystocia in the last days before parturition, compared to cows with normal parturition. ^2^ Performance herein shown is referred only to machine-learning analysis of data from SHM collar and for Activity parameter, which best fitted as calving predictor.

**Table 2:** Devices for automatic combined activity and feeding monitoring for calving prediction, with performance and references.

Parameter	Sensor Type	Device	N	Application	TI	Device Performance	Factory	References
Combination of activity, feeding, rumination and temperature	Accelerometer	RumiWatch	22 multiparous	Noseband sensor + pedometer	3 h	Multiparous cows	ITIN + HOCH GmbH, Fütterungstechnik CH-4410 Liestal, Switzerland	Fadul et al. [36]
						Se = 85%; Sp = 74%;	https://www.rumiwatch.com/	
						AUC = 90.8%		
			11 primiparous			Primiparous cows		
						Se = 88.9%; Sp = 93.3%;		
						AUC = 97.7%		
		RumiWatch	35	Noseband sensor	1 h	Se = 82%; Sp = 87%;	Agroscope, Ettenhausen, Switzerland and Itin + Hoch GmbH, Liestal, Switzerland	Zehner et al. [37]
						PPV = 4%; AUC = 82%	https://www.rumiwatch.com/	
		SensOor	42	Ear tag	24 h	Se = 51%; Sp = 51%;	Agis Automatisering BV, Harmelen, Netherlands	Ouellet et al. [29]
						PPV = 27%; NPV = 75%;	https://www.agis.nl/Cowmanager	
						AUC = 0.54		
					12 h	Se = 52%; Sp = 55%;		
						PPV = 15%; NPV = 88%;		
						AUC = 0.60		
					6 h	Se = 63%; Sp = 63%;		
						PPV = 11%; NPV = 95%;		
						AUC = 0.67		
			400		12 h	Se = 51.5%; Sp = 99.4%;		Rutten et al. [38]
						AUC = 90.1%		
					6 h	Se = 48.5%; Sp = 99.3%;		
						AUC = 90.1%		
					3 h	Se = 42.4%; Sp = 99.2%;		
						AUC = 90.1%		
					1 h	Se = 21.2%; 99.1%;		
						AUC = 90.1%		
		Smartbow^®^	150	Ear tag	24 h	Se = 27%; Sp = 96%;	Smartbow GmbH, Weibern, Austria	Krieger et al. [39]
			(validation set)			Acc = 92%	https://www.smartbow.com/	Krieger et al. [40]
			450					Krieger et al. [41]
			(validation set)					
			12 h	Se = 35%; Sp = 95%;				
				Acc = 94%				
					6 h	Se = 43%; Sp = 95%;		
						Acc = 94%		
					3 h	Se = 49%; Sp = 95%;		
						Acc = 94%		
					1 h	Se = 54%; Sp = 95%;		
						Acc = 94%		
			54		4 h ^1^	PPV = 12.6%		Horvàth et al. [31]
		Smartbow^®^	5	Ear tag (fixed to the tail)	from 6 to	n.a.	Smartbow GmbH, Weibern, Austria	Horvàth et al. [31] Krieger et al. [41]
					121 min		https://www.smartbow.com/	
		RT-BT-9axisIMU	3	Collar	n.a.	n.a.	RT Corporation, Tokyo, Japan	Peng et al. [42] Peng et al. [42]
							(NS)	

N: number of animals; NS: not specific, means that devices was self-assembled by researchers starting from data logger and sensor available in the market. This means that there are no commercially available products specific for use in cow. TI: time interval between calving alarm and parturition. Se: sensitivity; Sp: specificity; Acc: accuracy; PPV: positive predictive value; NPV: negative predicting value; AUC: area under curve; n.a.: not available. ^1^ Compared retrospectively to fetal sacs rupture, as identified by an intravaginal device.

**Table 3:** Devices for automatic temperature monitoring for calving prediction, with performance and references.

Parameter	Sensor Type	Device	N	Application	TI	Device Performance	Factory	References
Temperature	Rumen temperature	SmartStock	30	Rumen bolus	48–24 h	n.a.	SmartStock, LLC, Pawnee, OK https://www.smartstock-usa.com	Cooper-Prado et al. [43]
		Phase IV	266	Rumen bolus	24 h	Cut-off = −0.2 °C Se = 68–69%; Sp = 67–69%; AUC = 73–74%	Fase IV Ingegneria, Boulder, CO https://www.phaseivengr.com/solutions-demos/animal-health-identification/	Costa et al. [44]
					12 h	Cut-off = −0.2 °C Se = 69–70%; Sp = 64%; AUC = 71–72%		
	Rumen temperature and pH	SmaXtec	10	Rumen bolus	Eutocic delivery −0.48 °C at 20 h	n.a.	Animal Care GmbH, Graz, Austria https://smaxtec.com/en/	Kovàcs et al. [45]
			8		Dystocic delivery −0.23 °C at 32 h			
	Vaginal temperature	Minilog II-t	42	Vaginal canal	24 h	Se = 74%; Sp = 74%; PPV = 51%; NPV = 89%; AUC = 0.80	Vemco Ltd., Halifax, Canada https://support.vemco.com/s/ (NS)	Ouellet et al. [29]
					12 h	Se = 69%; Sp = 69%; PPV = 26%; NPV = 93%; AUC = 0.74		
					6 h	Se = 68%; Sp = 67%; PPV = 13%; NPV = 97%; AUC = 0.68		
	Tail base temperature	Thermistor Prototype: 103JT-025	35 22	Ventral tail surface	24 h	Se = 80–89%; Sp = 89–91%; PPV = 19–20%; NPV = 99%	SEMITEC Corporation, Tokyo, Japan http://www.semitec.co.jp/english/ (NS)	Koyama et al. [46], Miwa et al. [47]
					18 h	Se = 83–92%; Sp = 87–88%; PPV = 55–56%; NPV = 97–98%		
					12 h	Se = 84–90%; Sp = 82–85%; PPV = 35–38%; NPV = 98–99%		
					6 h	Se = 83–90%; Sp = 79–82%; PPV = 19–20%; NPV = 99%		
			108 (validation set)	Ventral tail surface + machine learning	24 h	Se = 84.3%; Pr = 70.5%		Higaki et al. [48]
	Vaginal temperature	Gyuonkei	625	Vaginal canal	~22 h	n.a.	Remote Inc., Oita, Japan http://www.gyuonkei.jp/	Sakatani et al. [49]
	Vaginal temperature	Vel’Phone^®^	211	Vaginal canal	24 h	Cut off = 38.2 °C Se = 86%; Sp = 91%; PPV = 80%; NPV = 88%; AUC = 0.89 Cut off = −0.21 °C Se = 66%; Sp = 76%; PPV = 67%; NPV = 69%; AUC = 0.72	Medria, Châteaugiron, France https://www.medria.fr/en/solutions/velphone/	Ricci et al. [50]

N: number of animals; NS: not specific, means that devices was self-assembled by researchers starting from data logger and sensor available in the market. This means that there are no commercially available products specific for use in cow. TI: time interval between calving alarm and parturition. Se: sensitivity; Sp: specificity; Acc: accuracy; PPV: positive predictive value; NPV: negative predicting value; AUC: area under curve; n.a.: not available.

**Table 4:** Tail movement sensor for calving prediction, with performance and references.

Event	Sensor Type	Device	Application	N	TI	Device Performance	Factory	References
Tail movement and raising	Accelerometer/inclinometer	Moocall	Tail base	12	24 h to 3 h	Se = 100%; Sp = 95% Se = 94%; Sp = 77%	Moocall Ltd., Dublin, Ireland https://www.moocall.com/	Giaretta et al. [51]
				118 *	24 h	Se = 75%; Sp = 63%; PPV = 56%; NPV = 79%		Voß et al. [52] ^1^
					12 h	Se = 69%; Sp = 74%; PPV = 44%; NPV = 89%		
					4 h	Se = 66%; Sp = 89%; PPV = 34%; NPV = 97%		
					2 h	Se = 43%; Sp = 93%; PPV = 21%; NPV = 97%		
					1 h	Se = 19%; Sp = 96%; PPV = 9%; NPV = 98%		
				54	4 h ^1^	PPV = 12.6%		Horvàth et al. [31]

N: number of animals. TI: time interval between calving alarm and parturition. Se: sensitivity; Sp: specificity; PPV: positive predictive value; NPV: negative predicting value. * = a total of 180 animals were involved in the study but only for 118 the sensor was already in use at the moment of calving. ^1^ Data herein reported are referred only to the “HTA1h” alert, that is detection of high tail activity in the previous hour.

**Table 5:** Devices for calving alarm which identify the onset of stage II of parturition, with performance and references.

Event	Sensor Type	Device	N	Application	TI	Device Performance	Factory	References
Vulvar lips separation	Magnetic sensor	Foalert, C6 birth control	22 80 53	Vulva (suture)	0 h	Se = 100; PPV = 95%	Sisteck Srl, Sassuolo, Italy https://www.foalingalarm.net/	Paolucci et al. [53] Paolucci et al. [54] Marchesi et al. [55]
	Magnetic sensor and GPS collar	GPS-CAL	26	Vulva (suture) + neck collar (GPS)	0 h	Se = 100%; PPV = 100%	Sisteck Srl, Sassuolo, Italy SiRF Technology, San Jose, California, USA	Calcante et al. [56]
Device expulsion	Light and temperature	OraNasco^®^	120 15 117 83	Vagina	0 h	Se = 86.30%	Kronotech Srl, Campoformido, Italy https://www.oranasco.it/	Palombi et al. [57] Rossi et al. [58] Crociati et al. [59] Crociati et al. [60]
	Light	iVET^®^	167	Vagina	0 h	Se = 78%; Sp = 93%	iVET^®^-Geburtsüberwachung für Kühe https://www.nrw-agrar.de/projekt/piloterprobung-des-geburtssensors-ivet-bei-milchkuehen-geburtsueberwachung/	Henningsen et al. [61]
	Temperature	Gyuonkei	625	Vagina	0 h	n.a.	Remote Inc., Oita, Japan http://www.gyuonkei.jp/	Sakatani et al. [49]
	Temperature	Vel’Phone^®^	211 241	Vagina	0 h	n.a.	Medria, Châteaugiron, France https://www.medria.fr/en/solutions/velphone/	Ricci et al. [50] Choukeir et al. [62]
			54		0 h	PPV = 100%		Horvàth et al. [31]

N: number of animals. TI: time interval between calving alarm and parturition. Se: sensitivity; Sp: specificity; Acc: accuracy; PPV: positive predictive value; NPV: negative predicting value; AUC: area under curve; n.a.: not available.

## Data Availability

Not applicable.
